# Molecular alterations associated with metastases of solid pseudopapillary neoplasms of the pancreas

**DOI:** 10.1002/path.5180

**Published:** 2018-11-27

**Authors:** Eliana Amato, Andrea Mafficini, Kenichi Hirabayashi, Rita T Lawlor, Matteo Fassan, Caterina Vicentini, Stefano Barbi, Pietro Delfino, Katarzyna Sikora, Borislav Rusev, Michele Simbolo, Irene Esposito, Davide Antonello, Antonio Pea, Elisabetta Sereni, Maria Ballotta, Laura Maggino, Giovanni Marchegiani, Nobuyuki Ohike, Laura D Wood, Roberto Salvia, Günter Klöppel, Giuseppe Zamboni, Aldo Scarpa, Vincenzo Corbo

**Affiliations:** ^1^ ARC‐Net Research Centre University and Hospital Trust of Verona Verona Italy; ^2^ Department of Diagnostics and Public Health, Section of Pathology University and Hospital Trust of Verona Verona Italy; ^3^ Department of Pathology Tokai University School of Medicine Isehara Japan; ^4^ Institute of Pathology, Heinrich‐Heine‐University and University Hospital of Düsseldorf Düsseldorf Germany; ^5^ Department of Surgery, General Surgery B University of Verona Verona Italy; ^6^ Section of Anatomic Pathology Azienda Ospedaliera Rovigo Rovigo Italy; ^7^ Department of Pathology and Laboratory Medicine Showa University Fujigaoka Hospital Yokohama Japan; ^8^ Department of Pathology The Sol Goldman Pancreatic Cancer Research Center, The Johns Hopkins University School of Medicine Baltimore MD USA; ^9^ Department of Pathology Technical University Munich Munich Germany; ^10^ Division of Pathology Sacro Cuore‐Don Calabria Hospital Negrar Italy

**Keywords:** Solid pseudopapillary neoplasms, pancreas, hypoxia, epigenetic regulators, metastasis

## Abstract

Solid pseudopapillary neoplasms (SPN) of the pancreas are rare, low‐grade malignant neoplasms that metastasise to the liver or peritoneum in 10–15% of cases. They almost invariably present somatic activating mutations of *CTNNB1*. No comprehensive molecular characterisation of metastatic disease has been conducted to date. We performed whole‐exome sequencing and copy‐number variation (CNV) analysis of 10 primary SPN and comparative sequencing of five matched primary/metastatic tumour specimens by high‐coverage targeted sequencing of 409 genes. In addition to *CTNNB1‐*activating mutations, we found inactivating mutations of epigenetic regulators (*KDM6A*, *TET1*, *BAP1*) associated with metastatic disease. Most of these alterations were shared between primary and metastatic lesions, suggesting that they occurred before dissemination. Differently from mutations, the majority of CNVs were not shared among lesions from the same patients and affected genes involved in metabolic and pro‐proliferative pathways. Immunostaining of 27 SPNs showed that loss or reduction of KDM6A and BAP1 expression was significantly enriched in metastatic SPNs. Consistent with an increased transcriptional response to hypoxia in pancreatic adenocarcinomas bearing *KDM6A* inactivation, we showed that mutation or reduced KDM6A expression in SPNs is associated with increased expression of the HIF1α‐regulated protein GLUT1 at both primary and metastatic sites. Our results suggest that BAP1 and KDM6A function is a barrier to the development of metastasis in a subset of SPNs, which might open novel avenues for the treatment of this disease. © 2018 The Authors. *The Journal of Pathology* published by John Wiley & Sons Ltd on behalf of Pathological Society of Great Britain and Ireland.

## Introduction

Solid pseudopapillary neoplasm (SPN) of the pancreas is a rare low‐grade malignant neoplasm that usually occurs in young women 1. Histologically, SPN is composed by polygonal cells that form a solid and pseudopapillary pattern, where they become discohesive. SPN has a peculiar immunophenotype among primary pancreatic neoplasms, which encompasses the expression of the mesenchymal marker vimentin, the protease inhibitor α1‐anti‐trypsin, the neuroendocrine marker neuron‐specific enolase 1, progesterone receptors, CD10, CD56, cyclin D1 and claudins 5 and 7 2. There may be some variable expression of synapthophysin and focal, faint positivity for cytokeratins 1.

The majority of SPNs are confined to the pancreas, with metastases to the liver and peritoneum in up to 15% of cases 1. SPN has an indolent clinical behaviour even for cases of large tumour size, and long‐term prognosis following surgical resection is generally excellent for both localised and distant disease 3.

Transcriptional activation of the WNT signalling pathway through the oncogenic mutation of *CTNNB1* (β‐catenin gene) is the major driver of SPN tumourigenesis 1. Mutations of *CTNNB1* clustering into exon 3 of the gene prevent degradation of the encoded protein β‐catenin, which accumulates in the nucleus to form transcriptionally active complexes with the DNA‐binding proteins TCF and LEF1 4.

In keeping with this, abnormal cytoplasmic and nuclear β‐catenin accumulation is evidenced by immunohistochemistry (IHC) in virtually all SPNs 5. In the nucleus, β‐catenin activates transcription of *CCND1* that is overexpressed in about 70% of SPNs 6. Whole‐exome sequencing (WES) of eight primary SPNs confirmed that they have nearly universal mutation of *CTNNB1* and demonstrated that these tumours have a low mutational burden and infrequent copy‐number changes compared with pancreatic adenocarcinoma 7.

Given that no molecular characterisation of metastatic SPN has been conducted to date, the genetic determinants of progression and metastasis of this tumour type remain undetermined. In this study, we undertook a comprehensive molecular characterisation of 10 primary SPNs for mutations and copy‐number variations (CNVs) of coding genes. In addition, we analysed five matched primary/metastatic lesions, performing high‐coverage targeted sequencing (HCTS) of the relevant regions of 409 cancer‐related genes to uncover genetic alterations involved in the progression of the disease.

## Materials and methods

### Human research ethical approvals

Ethics committee approval was obtained at the three institutions involved in the collection of SPN cases. ARC‐Net, University of Verona, Italy: approval number 1885 from the Integrated University Hospital Trust Ethics Committee (Comitato Etico Azienda Ospedaliera Universitaria Integrata). Tokai University, Japan: The Research Ethics Committee of Tokai University School of Medicine, Japan: approval number 17R275. University of Munich, Germany: approval number 503/16s from the Research Ethics Committee of Technical University of Munich.

### SPN cohort

A cohort of 27 treatment‐naive and surgically resected SPNs was included in this study; details of molecular analyses conducted on each case are reported in supplementary material, Supplementary materials and methods and Table S1.

All cases were classified according to World Health Organization 2010 criteria 1. The sequencing cohort included 15 cases whose clinicopathological information is provided in Table 1, comprising 11 with fresh‐frozen and four with formalin‐fixed paraffin‐embedded (FFPE) tissue available. Five of these SPNs were metastatic (one fresh‐frozen and four FFPE) and their matched liver metastases were available.

**Table 1:** Summary of clinicopathological characteristics of the 15 patients whose SPN were subjected to sequencing

	Case	Age at diagnosis (years)	Sex (M/F)	Tissue origin	Diagnosis	Primary tumour	Liver metastasis			Mitosis (10HPF)	Ki67 index (%)	Tumour necrosis*	Ischaemic necrosis*	Vascular invasion*	Perineural invasion*	Adipose tissue infiltration*	Lymph nodes*	Pleomorphism*
						Size (cm)	Size (cm)		Onset									
Non ‐metastatic tumours	SPN1	32	F	Pancreas	SPN	9	—	—	—	0	0	0	0	0	0	0	0	Light atypia†
	SPN2	25	F	Pancreas	SPN	8	—	—	—	0	0	0	0	0	0	0	0	0
	SPN3	50	F	Pancreas	SPN	3	—	—	—	0	0	0	0	0	1	1	0	0
	SPN4	40	F	Pancreas	SPN	5	—	—	—	0	0	0	0	0	0	0	0	0
	SPN5	17	M	Pancreas	SPN	3.5	—	—	—	0	0	0	0	0	0	0	0	0
	SPN6	43	F	Pancreas	SPN	4.5	—	—	—	0	0	0	0	0	0	0	0	0
	SPN7	21	F	Pancreas	SPN	3	—	—	—	0	0	0	0	0	0	0	0	0
	SPN8	12	F	Pancreas	SPN	14	—	—	—	0	0	0	0	0	0	0	0	0
	SPN9	17	F	Pancreas	SPN	7	—	—	—	0	0	0	0	0	0	0	0	0
	SPN10	24	F	Pancreas	SPN	5	—	—	—	0	0	0	0	0	0	0	0	0
Metastatic tumours	SPN11	37	F	Pancreas	SPN	5				0	<1	0	1	0	0	0	0	0
	SPN11_L	37		Liver	Metastasis ofSPN		6	Multiple	Synchronous	0	<1	0	1	1	0	0	0	0
	SPN12	14	F	Pancreas	SPN	9				0	<1	0	1	0	0	0	0	0
	SPN12_L			Liver	Metastasis ofSPN		1.3	Solitary	30 months		<1							
	SPN13	36	F	Pancreas	SPN	9				12	14	1	0	1	0	1	0	1
	SPN13_La			Liver	Metastasis ofSPN		3.5	Multiple	9 months	20	80	1	0	1	0	0	0	1
	SPN13_Lb			Liver	Metastasis ofSPN		1.2			15	49.1	1	0	0	0	0	0	0
	SPN13_Lc			Liver	Metastasis ofSPN		1.8			20	74.8	1	0	1	0	0	0	1
	SPN14	63	F	Pancreas	SPN	10				15	19.8	0	1	1	1	1	0	0
	SPN14_La			Liver	Metastasis ofSPN		13	Multiple	Synchronous and recurrence		17.6							
	SPN14_Lb			Liver	Metastasis ofSPN													
	SPN56	23	F	Pancreas	SPN	8.5				0	<3	1	0	1				1
	SPN56_L			Liver	Metastasis ofSPN		1.5	Multiple	Synchronous	6	3	0	0					1
																	

Blank fields, not evaluable.

* 0, absent; 1, present.

† Defined as the presence of mild hyperchromasia and increased nuclear groves.

### Immunohistochemistry

The following primary antibodies were used according to described staining protocols: β‐catenin (Sigma‐Aldrich, Milan, Italy; clone 15B8, 1:400 dilution) 2, GLUT1 (Bio‐Optica, Milan, Italy; clone RB‐9052, 1:100 dilution) 8, BAP1 (Santa Cruz Biotechnology, Heidelberg, Germany; clone C‐4, 1:100 dilution) 9, KDM6A (Cell Signalling Technology, Leiden, The Netherlands; clone D3Q1l, 1:200 dilution) 10, p53 (Novocastra‐Leica, Buccinasco, Milan, Italy; clone DO‐7, 1:50 dilution) 11. BTD immunostaining was performed using a polyclonal antibody (Thermo Fisher Scientific, Monza, Milan, Italy; PA5‐28180, 1:500 dilution) and standard IHC procedures 12; endothelial cells served as internal positive controls for this antigen. For β‐catenin, sections were evaluated for the presence of nuclear, cytoplasmic and membranous staining. Abnormal β‐catenin immunostaining was defined as nuclear accumulation of the protein. Membranous and cytoplasmic immunostaining was considered for GLUT1 13. Cytoplasmic staining was considered for BTD, whereas nuclear staining was evaluated for both KDM6A and BAP1. Immunostaining for BAP1 was defined as ‘negative’ or ‘positive’, without considering an intensity cut‐off value, due to unambiguous absence or presence of nuclear staining; specimens were classified as heterogeneous when at least 25% of neoplastic cells showed no nuclear staining. A three‐tier intensity score (1+, 2+, 3+) was used for KDM6A and GLUT1 to denote weak, moderate and strong staining intensity, respectively. In particular, ‘strong’ for GLUT1 was defined as the staining intensity observed in red blood cells, whereas ‘strong’ and ‘weak’ for KDM6A were defined as the staining intensity showed by islets or acinar cells from non‐neoplastic pancreas, respectively. For data analysis, cases were classified as either ‘high’ or ‘low’ by combining intensity scores as follow: 0 and 1+ (low); 2+ and 3+ (high).

### Sequencing analysis

WES was performed on 10 primary tumour/normal DNA pairs using the SOLID 4 platform (Thermo Fisher Scientific). An additional case, for which a matched liver metastatic lesion was available, was subjected to WES using the HiSEQ 2000 platform (Illumina, San Diego, CA, USA). Mutational and CNV analyses were performed as described in supplementary material, Supplementary materials and methods.

### Genome‐wide copy‐number analysis

Genome‐wide SNP genotyping was performed on 10 primary tumours with the Illumina GoldenGate assay (Illumina). DNA copy‐number changes were estimated using the ASCAT software release 2.1 14.

### Targeted sequencing and copy‐number analysis

Matched primary/metastatic lesions for a total of 13 samples (five primaries and eight metastases) were sequenced using the Ion Ampliseq Comprehensive Cancer Panel (Thermo Fisher Scientific), which targets the relevant regions of 409 genes. The complete gene list of this assay and details of the targeted regions can be found at http://www.thermofisher.com. Data analysis of HCTS and orthogonal validation of mutations were performed as described in supplementary material, Supplementary materials and methods.

### Computational and statistical analysis

Statistical analyses were conducted using Prism5 (GraphPad Software, La Jolla, CA, USA), and *p* < 0.05 was considered statistically significant. Fisher's exact test, chi‐squared test and chi‐squared test for trend were used as appropriate to assess the significance of BAP1, KDM6A and GLUT1 status and disease progression in the IHC analysis of SPN tissues. For data mining, we used the National Cancer Institute's Genomic Data Commons portal (https://portal.gdc.cancer.gov/) and the International Cancer Genome Consortium portal (http://icgc.org/). To determine the enrichment of genes involved in response to hypoxia in pancreatic ductal adenocarcinoma (PDAC) with inactivation of *KDM6A*, we used the R/Bioconductor package GSVA 15 and the MSigDB_HALLMARK_HYPOXIA dataset containing 200 genes. GSVA enrichment scores were generated using transformed count data and stratified based on *KDM6A* status. The Wilcoxon rank‐sum test was applied to the stratified scores to compare normal and mutated samples.

## Results

The study workflow is illustrated in supplementary material, Figure S1. For sequencing analysis, 15 SPN cases were collected. Of these 15 cases, five had metastases to the liver that were also available for sequencing analysis. Clinicopathological characteristics of the SPNs of the sequencing cohort are provided in Table 1. IHC analyses were conducted on the specimens from the sequencing cohort and on 12 additional SPNs, comprising 10 non‐metastatic and two metastatic cases (see supplementary material, Table S1). Matched tumour/normal DNA of 10 non‐metastatic SPNs underwent WES and high‐density SNP array (see supplementary material, Tables S2 and S3). Comparative lesion sequencing 16, 17 was performed by WES on one metastatic case with a single metastasis to the liver (see supplementary material, Tables S2 and S3). HCTS of the relevant region of 409 cancer‐related genes was performed on the above case and four additional matched primary/metastatic specimens (see supplementary material, Table S4).

### Clinicopathological characteristics of primary and metastatic SPNs

Most primary tumours showed the classical SPN microscopic features characterised by a heterogeneous combination of solid and cystic components. The solid components consisted of pseudopapillae with vascular stalks and hyalinised stroma within tumour areas and were intermingled by cystic haemorrhagic areas (see supplementary material, Figure S2A,B). Two of the metastatic tumours showed a diffuse solid growth pattern with minimal supporting fibrovascular stroma (see supplementary material, Figure S2C). This microscopic appearance was also maintained in the metastatic disease. Both of these tumours showed morphological features of aggressive behaviour, including mitoses, increased nuclear to cytoplasmic ratio, nuclear hyperchromasia and mild cellular atypia. In one of these two cases (SPN13), one of three hepatic lesions also presented with a distinct morphological component having sarcomatoid/dedifferentiated aspects (see supplementary material, Figure S3).

### Somatic mutations and copy‐number changes in non‐metastatic SPNs

**Figure 1:**
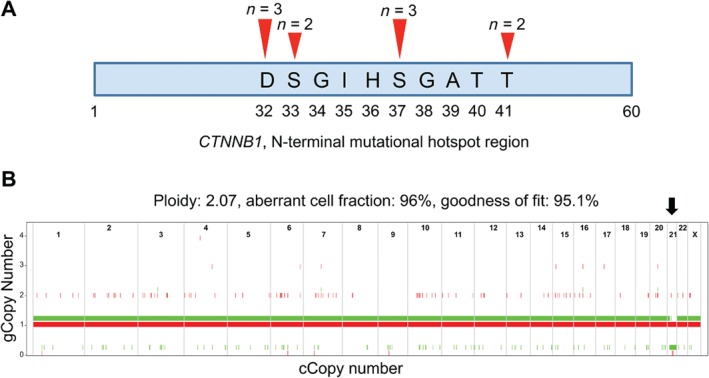
Somatic alterations identified in 10 primary SPNs. (A) Somatic mutations in *CTNNB1* are missense mutations that cluster into exon 3 of the gene. The first 60N‐terminal amino acids are represented. (B) LOH at chromosome 21 in case SPN3. The analysis shows Illumina GoldenGate assay (Illumina) SNP Array data from matched tumour and non‐malignant DNA, with ASCAT estimates of the genomic copy number of the two parental copies of each chromosome (arbitrarily coloured in red and green). The genomic copy number is shown along the *y*‐axis, while the chromosomal copy number is shown along the *x*‐axis. In SPN3, ASCAT estimated that one copy of chromosome 21 (the ‘green’ copy) is completely deleted (copy number 0), leading to LOH (black arrow).

WES was performed on 10 primary SPNs and achieved a mean coverage of 44× in the tumour and 34× in matched normal samples. Detailed coverage information and the percentage of targeted bases are reported in supplementary material, Table S2. Activating mutations of *CTNNB1* were present in all SPNs and were the only non‐synonymous coding mutations identified in our cohort (see supplementary material, Table S3). All 10 *CTNNB1* mutations were 1‐bp missense mutations affecting exon 3 of the gene, distributed as follows: three mutations at codon 32, three at codon 37, two at codon 33 and two at codon 41 (Figure 1A). The allele frequency of *CTNNB1* mutations was in the range of 21–44% (see supplementary material, Table S3). Copy‐number analysis revealed that all cases were diploid except for one tumour (SPN3) showing loss of heterozygosity (LOH) on chromosome 21 (Figure 1B). Beyond mutations of *CTNNB1*, no recurrent genetic events were found in non‐metastatic SPNs.

### Somatic mutations in metastatic SPN

**Figure 2:**
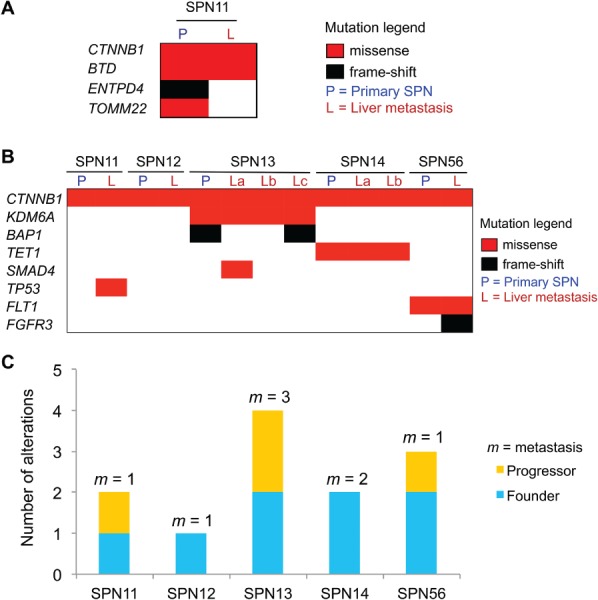
Somatic alterations in metastatic SPNs of the pancreas. (A) Somatic mutations identified in primary tumour and matched metastatic lesion of the index case by WES analysis. (B) Somatic mutations identified in matched primary/metastatic samples by targeted sequencing. (C) Total somatic mutations are displayed per case, including alterations shared among all lesions (founder) and those detected in one or more but not all of the specimens for a given case (progressor). The number of individual metastatic lesion (*m*) sequenced per case is indicated. See supplementary material, Table S4 for details.

To address whether alterations other than *CTNNB1* mutations are associated with the metastasis of SPN, we performed WES of an index case (SPN11), for which frozen tissue of a liver metastatic lesion was also available. WES achieved coverage of 60× in the tumour, 71× in the metastatic lesion and 21× in the normal sample (see supplementary material, Table S2). A total of four and two non‐synonymous mutations were found in the tumour and the metastatic sample, respectively (Figure 2A). Mutations in *CTNNB1* (p.D32Y) and in the histones biotinylation gene *BTD* 18 (p.L297Q) were shared between tumour and metastasis, whereas mutations in the apyrase gene *ENTPD4* (p.R340NfsTer14) and the translocase of outer mitochondrial membrane gene *TOMM22* (p.V99I) were identified exclusively in the primary. Mutations in *BTD* and *ENTPD4* were predicted to be deleterious for gene function, whereas mutation in *TOMM22* (p.V99I) was predicted to be benign (see supplementary material, Table S3). To further confirm that progression of SPN is associated with accumulation of genetic alterations and to explore the clonal relationship between primaries and metastases, we performed HCTS of the relevant regions of 409 cancer‐related genes in four additional primary/metastatic specimens for which only FFPE tissues were available. The index case was also included in this analysis, leading to a total of 13 specimens analysed (five primaries and eight metastatic lesions). HCTS yielded an average coverage of 100× for at least 70% of targeted bases in both neoplastic and non‐neoplastic samples. In total, 27 mutations affecting eight genes, which included *CTNNB1*, *KDM6A*, *BAP1*, *TET1*, *SMAD4*, *TP53*, *FLT1* and *FGFR3* (Figure 2B), were identified. Almost all mutations were missense, except for frameshift deletions affecting the genes encoding for the deubiquitinating enzyme BAP1 and the tyrosine kinase receptor FGFR3 (see supplementary material, Table S4). Mutations identified through WES in the index case could not be confirmed by HCTS, as the affected genes (*BTD*, *ENTPD4*, *TOMM22*) were not available in the targeted panel. Germline mutations of *BTD* cause an autosomal recessive disorder that can lead to delayed neurological development 19. However, the role of this gene in cancer as well as the expression of its protein product in normal and neoplastic pancreatic tissues are neglected to date. Therefore, we first assessed the expression of BTD by IHC and found no immunoreactivity in both normal pancreas and in the 27 SPNs analysed. BTD staining was instead prominent in endothelial cells and normal hepatocytes (see supplementary material, Figure S4A). We concluded that the mutation of *BTD* was probably a passenger event. A low allele frequency (4%) missense mutation of *TP53* was identified through HCTS of the metastatic specimen from the index case. Consistently, p53 immunoreactivity in this case was limited to a few nuclei (see supplementary material, Figure S4B), whereas none of the other metastatic or non‐metastatic specimens showed any immunostaining. In keeping with our observation, mutation of *TP53* has been previously reported as a rare event in SPNs 11. As expected for a founder mutation, *CTNNB1* alterations were shared among primary and matched metastatic lesions. Additional events that were present in all samples from a given patient included *KDM6A* mutations in SPN13, *TET1* mutations in SPN14 and *FLT1* mutations in SPN56. Mutations present in one or more but not all specimens from a given patient, and as such defined as progressor mutations, affected *BAP1* and *SMAD4* in SPN13 and *FGFR3* in SPN56. In particular, *BAP1* mutation was detected in the primary tumour and only in one of the three liver lesions, whereas *SMAD4* mutation was detected exclusively in one metastatic lesion but not in the primary tumour. These data suggest that the majority of mutations occur before metastatic spread as they are shared among the different lesions from a given patient (Figure 2C). However, the identification of lesion‐specific mutations indicates that subclones emerged either independently at primary or at metastatic sites as a consequence of ongoing clonal evolution.

### Copy‐number changes in metastatic SPN

**Figure 3:**
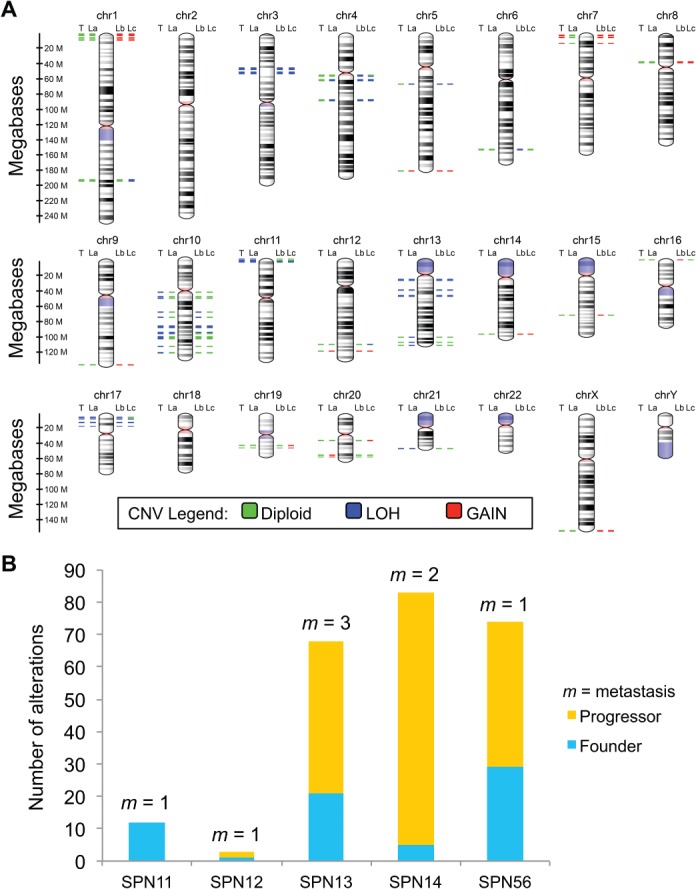
Somatic copy‐number changes in metastatic SPN of the pancreas. (A) The virtual karyotype view shows the location, proximity and copy‐number status of altered genes in the most representative case (SPN13) with the primary and three matched metastatic lesions available. The colouring scheme of chromosomal bands is as follows: black and grey = Giemsa positive; light red = centromere; purple = variable region. Alterations are annotated according to the colour codes presented in the figure. P, primary SPN; L(a–c), liver metastases. (B) Total somatic alterations (genes affected by CNV) are displayed per case, including alterations shared among all lesions (founder) and those detected in one or more (but not all) of the specimens for a given case (progressor). The number of individual metastatic lesion (*m*) sequenced per case is indicated. See supplementary material, Figure S5 and Table S5 for details.

CNV analysis at the chromosomal level was feasible only for the index case (SPN11) using WES data (supplementary material, Table S3). LOH of chromosome 22 detected through BAF analysis in the primary tumour was shared by the matched metastatic sample (see supplementary material, Table S3). Gene‐level CNV analysis of the five metastatic SPN cases was conducted using HCTS data and revealed alterations in all the specimens analysed (see supplementary material, Figure S5). Figure 3A shows the detail of CNV differences detected among the four lesions analysed for the case SPN13. Differently from mutations, many CNVs were not shared among different specimens from a given case, thereby suggesting that they were independently acquired in each lesion as contributors to the progression of the disease (Figure 3B). Pathway enrichment analysis of genes with losses and gains among the SPN cases (SPN11, SPN13, SPN14 and SPN56) showed that altered genes are involved in metabolic and pro‐proliferative pathways, such as central carbon metabolism, Rap1, Ras, Pi3K/Akt, mTOR and focal adhesion, all of which are known to be involved in tumour progression 20, 21, 22, 23, 24, 25 (see supplementary material, Tables S6A,B).

### Inactivation of epigenetic regulators in metastatic SPNs

**Figure 4:**
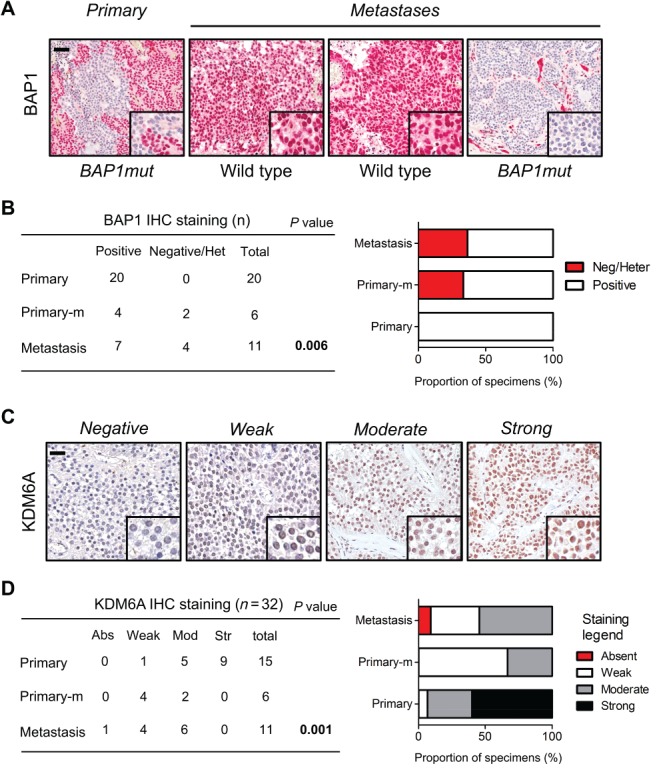
Expression of BAP1 and KDM6A in primary SPN of the pancreas and in liver metastases. (A) BAP1 expression was evaluated by immunostaining in primary tumour and matched metastatic lesions. BAP1mut denotes specimens bearing inactivating mutation of the gene, whereas wild type denotes specimens lacking detectable mutations. (B) Contingency table (left) and stacked bar graph (right) showing BAP1 staining intensity in non‐metastatic and metastatic SPNs. Primary‐m, primary metastatic tumour; Het, heterogeneous staining as defined in supplementary materials, Supplementary materials and methods and Table 2. Increase in alteration from primary to metastasis was determined by chi‐squared test for trend; scale bar is 100 μm and inset magnification 600×. (C) Representative IHC images showing different staining intensity for KDM6A in SPNs. Scale bar is 100 μm and inset magnification 600×. (D) Contingency table (left) and stacked bar graph (right) showing KDM6A staining intensity in non‐metastatic and metastatic SPNs. Abs, absent; Mod, moderate; Str, strong. Primary‐m, primary metastatic tumour. Significance was determined by Fisher's exact test.

Two of the five metastatic cases in our cohort showed mutations that were predicted to inactivate genes involved in epigenetic regulation, namely *BAP1*, *KDM6A* and *TET1*. Mutations of *BAP1* and *KDM6A* were concurrent in the primary and one metastatic lesion of case SPN13, and co‐occurring mutations of these genes have already been reported in bladder cancer 26. KDM6A (also known as UTX) is a core component of the ‘complex of proteins associated with SET1’ (COMPASS) 10, 27 and it has been shown to require BAP1 for its recruitment at chromatin enhancers 28. Inactivation of those genes has been reported to occur through different mechanisms, ranging from DNA methylation to complex DNA rearrangements 25, 29, which can be missed by targeted DNA sequencing. IHC is a reliable method to assess the status of BAP1 30, while IHC for KDM6A has been recently used to assess the status of KDM6A in PDAC 10. Therefore, we expanded the analysis of *BAP1* and *KDM6A* status to the entire cohort of 27 SPNs (20 primaries and seven metastatic cases) by performing IHC on FFPE tissues. BAP1 was expressed in both islets and exocrine pancreatic cells (see supplementary material, Figure S4C). The primary tumour and the matched liver metastasis of case SPN13 sharing a deleterious mutation of *BAP1* featured heterogeneous and homogenously negative staining for the gene product, respectively. Conversely, the other two hepatic metastases from SPN13 presented a homogeneously positive staining consistent with the absence of *BAP1* mutation (Figure 4A). *BAP1* was always expressed in the 20 non‐metastatic SPNs, whereas staining was heterogeneous in three metastatic SPNs (one primary tissue and two metastases) and uniformly negative in one metastatic lesion (Figure 4B, Table 2). An inverse correlation was observed between metastatic SPNs and BAP1 staining (Figure 4B). In agreement with a previous study 10, *KDM6A* was expressed in islets and presented a mosaic expression in exocrine pancreas (see supplementary material, Figure S4C). Specimens from the case SPN13 bearing a missense mutation of *KDM6A* showed moderate staining, suggesting that the genetic alteration, which occurred proximal to the enzymatic domain, should not affect protein expression, but rather its function (Table 2). Strong expression of *KDM6A* was only detected in non‐metastatic SPNs (Figure 4C, Table 2), whereas staining was absent in one hepatic lesion from a metastatic SPN. Overall, there was a statistically significant inverse correlation between metastatic SPNs and KDM6A staining (Figure 4C).

**Table 2:** Summary of BAP1and KDM6A IHC on SPNs of 27 patients

	Sample ID	Tissue origin	Diagnosis	IHC for BAP1	IHC for KDM6A
Non‐metastatic SPNs	SPN1	Pancreas	SPN	P	NE
	SPN2	Pancreas	SPN	P	NE
	SPN3	Pancreas	SPN	P	NE
	SPN4	Pancreas	SPN	P	NE
	SPN5	Pancreas	SPN	P	1
	SPN6	Pancreas	SPN	P	3
	SPN7	Pancreas	SPN	P	2
	SPN8	Pancreas	SPN	P	3
	SPN9	Pancreas	SPN	P	NE
	SPN10	Pancreas	SPN	P	3
	SPN46	Pancreas	SPN	P	2
	SPN47	Pancreas	SPN	P	2
	SPN48	Pancreas	SPN	P	2
	SPN49	Pancreas	SPN	P	3
	SPN50	Pancreas	SPN	P	3
	SPN51	Pancreas	SPN	P	3
	SPN52	Pancreas	SPN	P	3
	SPN53	Pancreas	SPN	P	3
	SPN54	Pancreas	SPN	P	2
	SPN55	Pancreas	SPN	P	3
Metastatic SPNs	SPN11	Pancreas	SPN	P	1
	SPN11_L	Liver	Metastasis of SPN	P	1
	SPN12	Pancreas	SPN	H	1
	SPN12_L	Liver	Metastasis of SPN	N	2
	SPN13	Pancreas	SPN	H	2
	SPN13_La	Liver	Metastasis of SPN	P	2
	SPN13_Lb	Liver	Metastasis of SPN	P	2
	SPN13_Lc	Liver	Metastasis of SPN	N	2
	SPN14	Pancreas	SPN	P	1
	SPN14_La	Liver	Metastasis of SPN	P	1
	SPN14_Lb	Liver	Metastasis of SPN	P	1
	SPN16_La	Liver	Metastasis of SPN	H	2
	SPN16_Lb	Liver	Metastasis of SPN	H	0
	SPN56	Pancreas	SPN	P	1
	SPN56_L	Liver	Metastasis of SPN	P	1
	SPN57	Pancreas	SPN	P	1
	SPN57_L	Liver	Metastasis of SPN	P	2

P, positive nuclear staining in 100% of tumour cells; N, negative for nuclear staining in 100% of tumours cells; H, heterogeneous nuclear staining with at least 25% of negative tumour cells; NE, not evaluable due to the absence of a positive internal control.

0, no tumour nuclear staining; 1, weak tumour nuclear staining; 2, moderate tumour nuclear staining; 3, strong tumour nuclear staining.

### Reduced expression of KDM6A is associated with upregulation of GLUT1 in SPN

**Figure 5:**
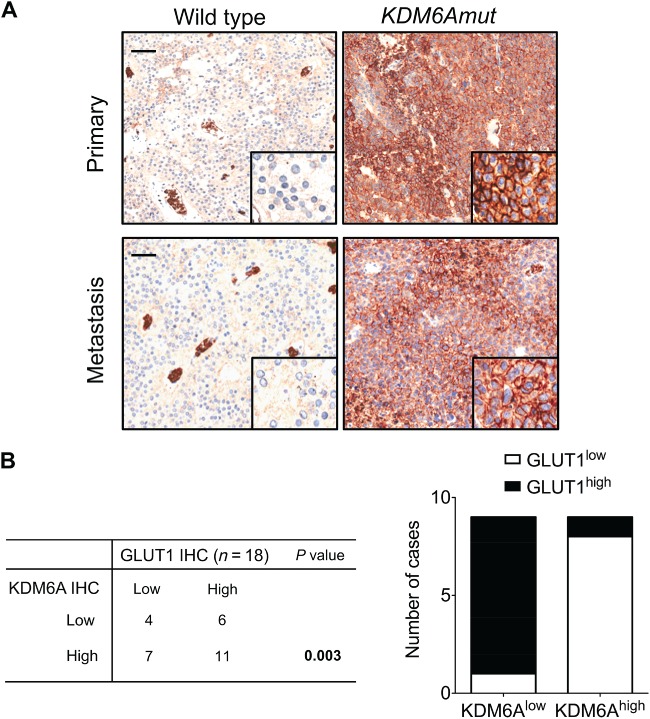
Expression of the hypoxia marker GLUT1 in primary SPN of the pancreas and in liver metastases. (A) Hypoxia was evaluated by immunostaining for GLUT1 in primary tumours (top panels) and liver metastases of SPNs (bottom panels) bearing mutations of KDM6A (KDM6Amut) or being wild type. Strong immunoreactivity for GLUT1 was observed in tissues from mutated SPNs, whereas no immunoreactivity was observed in tissues from wild type SPN. Positive staining for GLUT1 was observed in blood cells of wild type tissue and served as an internal positive control. Scale bars are 100 μm and inset magnification 600×. (B) Stacked bar graph showing an inverse correlation between expression of KDM6A and GLUT1 as assessed by IHC staining. For both GLUT1 and KDM6A, the category ‘low’ includes cases with absent or weak immunostaining, whereas ‘high’ denotes moderate or strong staining. Significance was determined by Fisher's exact test.

Direct and indirect evidence suggests that KDM6A is sensitive to changes in oxygen levels within the cells 31. Inactivation of KDM6A is enriched in aggressive tumours, including PDAC 32. Interrogating the International Cancer Genome Consortium (ICGC) cohort, we found that a hypoxia transcriptional signature is enriched in PDAC characterised by *KDM6A* inactivation (see supplementary material, Figure S6A). Although mechanisms are not always shared among tumour types, we sought to investigate whether mutation or reduced expression of *KDM6A* was also associated with a hypoxic signature in SPNs. To identify a reliable marker of hypoxia linked to *KDM6A* status, we retrieved PDAC data from the ICGC and The Cancer Genome Atlas (TCGA) databases and found that in both cohorts the hypoxia‐related gene *SCL2A1* (encoding for GLUT1) was significantly upregulated when KDM6A was inactive (see supplementary material, Figure S6B,C). Based on this information, we immunostained 18 samples from our cohort for GLUT1. GLUT1 expression was strongly upregulated in *KDM6A*‐mutated tumours (both primary and metastatic) compared with *KDM6A*‐proficient tumours (Figure 5A). Moreover, we found a trend to increased expression of GLUT1 in advanced SPNs (*p* = 0.1) and an inverse correlation between GLUT1 and KDM6A staining (*p* = 0.003, Figure 5B).

## Discussion

Here we describe for the first time the genetic events associated with progression and metastasis of SPN. In keeping with previous reports 4, 5, 7, we showed that activating mutation of *CTNNB1* is a universal feature of primary SPN and that no other recurrent alteration that affects protein‐coding genes could be identified. Moreover, we found that clinically benign SPNs have diploid genomes and no relevant CNV, whereas one case with features of malignancy, including cellular atypia and high proliferative index associated with necrosis, showed LOH on chromosome 21. Overall, our data suggest that transcriptional activation of the WNT pathway is sufficient to drive tumourigenesis of SPN. Metastasis is the most common cause of cancer‐related death 33. Although metastasis can occur in up to 15% of SPN patients 1, only a few patients harbouring an undifferentiated carcinoma component have died of a metastasising SPN 34. How cancer cells acquire the ability to colonise distant organs remains an unsolved question for SPN, mainly due to the paucity of metastatic specimens for molecular analyses. We used HCTS to analyse the clonal relationship between primary and hepatic lesions in five patients and found that the majority of mutations were shared among primaries and metastases, thereby suggesting that these events precede dissemination of cancer cells. Differently from mutations, the majority of CNVs were not shared among primaries and metastases. Although the reasons for this difference are difficult to infer from our data, we can speculate that differences in gene dosage reflect the selective pressure exerted by the different microenvironments or that subclones exist within primary tumours that could not be identified by our approach. Pathway enrichment analysis of genes affected by CNV in metastatic SPNs coherently identified biological processes known to sustain proliferation of cancer cells and metastatic behaviour. The majority of genetic alterations identified in metastatic SPNs affected *bona fide* tumour suppressor genes involved in epigenetic regulation of gene expression (*KDM6A*, *TET1* and *BAP1*). Of those, *BAP1* 35, 36, 37 and *KDM6A* 25, 32 are genes commonly inactivated in aggressive and metastatic tumours. BAP1 and KDM6A have been shown to interact at chromatin enhancers 28 and disruption of this functional interaction in cancers drives malignancy through a mechanism that can be pharmacologically targeted 28. Inactivation of those genes has been reported to occur through different genetic and epigenetic mechanisms 25, 29, which can be missed by targeted DNA sequencing approaches and often lead to the loss of functional proteins. In our cohort of SPNs, we found that a lack of or reduced expression of both *KDM6A* and *BAP1* is enriched in metastatic cases, suggesting that their function is a barrier to the development of metastatic disease at least in a subset of SPNs. *KDM6A* inactivation is also observed in PDAC 25, where it associates with an aggressive and metastatic molecular subtype driven by ‘squamous‐like’ transcriptional programmes, including response to hypoxia 38. In keeping with this, our interrogation of ICGC and TCGA databases revealed that a hypoxia transcriptional signature is upregulated in PDAC tumours with inactivation of *KDM6A*. We explored this signature and identified GLUT1 as a biomarker that links hypoxia to *KDM6A* status in PDAC, and leveraged this information to verify if similar mechanisms were operating in SPNs. Although expression of GLUT1 did not significantly discriminate between metastatic and non‐metastatic SPNs, we show here that tumours bearing inactivation of *KDM6A* or showing reduced protein expression strongly upregulate the expression of the hypoxia‐marker GLUT1 in both primary and metastatic tissues. Although tumour hypoxia is strongly associated with cancer progression and resistance to therapy, we cannot conclude whether in SPN mutation or reduced expression of *KDM6A* is an epiphenomenon of oxygen shortage or instead precedes the formation of a hypoxic microenvironment.

Despite our sequencing cohort representing the largest analysed to date, we could not identify recurrent genetic alterations in metastatic SPNs. This may be due to either the number of cases analysed or the targeted sequencing approach, which have possibly precluded us from identifying common drivers of SPN progression. Nevertheless, this study provides the most comprehensive description, to date, of the molecular events that characterise the progression of malignancy in SPN and indicates alterations of *BAP1* and *KDM6A* as potential drivers of metastasis in SPNs. This might have important therapeutic implication as, in other tumour types, *BAP1* loss and reduced expression of *KDM6A* have been shown to drive malignancy through epigenetic alterations that can be pharmacologically reverted 28. Although we have provided evidence that mechanisms can be shared between different malignancies, it remains to be determined whether *BAP1* and *KDM6A* alterations are promoting tumour growth in SPNs through a similar epigenetic mechanism. In the absence of preclinical models, this might only happen through multidimensional tumour profiling that will necessarily involve the integration of genetic and epigenetic analyses of a larger cohort of SPNs.

## Author contributions statement

AS, VC and GZ conceived the study. EA, AM, KH and RTL designed the study. EA, MS and DA designed the validation experiment. MF supervised the validation experiment. RTL co‐ordinated patients and sample data management and supervised ethical protocols. AP, LM, GM, ES, RS, MB, NO, IE and GK collected materials and clinical data. GZ, MF, BR, GK, MB, IE and AS analysed histopathological data. EA, SB, KS, DA and AM carried out deep sequencing and raw data analysis. AM, PD and SB performed bioinformatic analysis. MF, CV and VC analysed IHC. VC, EA, AM and MF drafted the manuscript. GK, RTL and LDW revised the manuscript. VC and AS finalised the paper. All authors approved the submitted version.

**SUPPLEMENTARY MATERIAL ONLINE**

**Supplementary materials and methods**

**Supplementary figure legends**

**Figure S1.** Flow charts of the sequencing analysis conducted on 154 SPN cases

**Figure S2.** Histological appearance of SPNs

**Figure S3.** Histological characteristics of metastatic SPN

**Figure S4.** IHC staining for BTD, TP53, KDM6A and BAP1

**Figure S5.** Detail of gene‐level somatic copy‐number changes in five metastatic SPNs of the pancreas

**Figure S6.** SLC2A1 expression is upregulated in PDAC bearing alterations of KDM6A

**Table S1.** Details of molecular analyses conducted on 27 SPNs

**Table S2.** WES summary data for 11 SPN specimens

**Table S3.** Somatic alterations identified in 11 SPN specimens by WES and SNP array analysis

**Table S4.** Somatic alterations identified in matched tumour/metastatic specimens by targeted sequencing of 409 genes

**Table S5.** Somatic gene copy‐number alterations identified in matched tumour/metastatic specimens by targeted sequencing of 409 genes

**Table S6A.** Enrichment analysis of main altered pathways according to copy‐number alterations in four metastatic SPNs

**Table S6B.** Detailed list of genes in main affected pathways according to copy‐number alterations analysis in four metastatic SPNs

## Supporting information


**Supplementary materials and methods**

**Supplementary figure legends**

**Figure S1.** Flow charts of the sequencing analysis conducted on 154 SPN cases

**Figure S2.** Histological appearance of SPNs

**Figure S3.** Histological characteristics of metastatic SPN

**Figure S4.** IHC staining for BTD, TP53, KDM6A and BAP1

**Figure S5.** Detail of gene‐level somatic copy‐number changes in five metastatic SPNs of the pancreas

**Figure S6.** SLC2A1 expression is upregulated in PDAC bearing alterations of KDM6A

**Table S1.** Details of molecular analyses conducted on 27 SPNs

**Table S2.** WES summary data for 11 SPN specimens

**Table S3.** Somatic alterations identified in 11 SPN specimens by WES and SNP array analysis

**Table S4.** Somatic alterations identified in matched tumour/metastatic specimens by targeted sequencing of 409 genes

**Table S5.** Somatic gene copy‐number alterations identified in matched tumour/metastatic specimens by targeted sequencing of 409 genes

**Table S6A.** Enrichment analysis of main altered pathways according to copy‐number alterations in four metastatic SPNs

**Table S6B.** Detailed list of genes in main affected pathways according to copy‐number alterations analysis in four metastatic SPNs
