# Supplementary material for: Molecular alterations associated with metastases of solid pseudopapillary neoplasms of the pancreas
Source: J Pathol. 2018 Nov 27;247(1):123–34. doi: 10.1002/path.5180 (PMC6588017; doi:10.1002/path.5180)
Supplement: Supplementary file 1 — Supplementary materials and methods [file PATH-247-123-s001.docx]

**Molecular alterations associated with metastases of solid pseudopapillary neoplasms of the pancreas**

Amato E *et al*. *J Pathol* 2018 (DOI: 10.1002/path.5180)

**Supplementary materials and methods**

Reference numbers refer to the main text list

**Tumour cell enrichment and DNA preparation**

Manual microdissection was applied when necessary to increase neoplastic cell content of the specimen to at least 70% before nucleic acid extraction. For SPN11, neoplastic cell enrichment achieved 55%. Uninvolved pancreatic tissue was used as a source of germline DNA to assess the somatic/germline nature of mutations.

**Sequencing and variant calling**

Reads generated with SOLID 4 were aligned using NovoalignCS Release 2.07.15 (Novocraft, Selangor, Malaysia) with human GRCh37/hg19 assembly (http://hgdownload.cse.ucsc.edu) as reference; the algorithm was used with its default setting excluding for the following parameters: (1) the threshold alignment score for acceptability was set at 180, (2) the thresholds for polyclonal filter was set as follow: reads were flagged as a low quality if either four of first 20 bp were below Q10 or 20% of all bases were below Q10, (3) paired end orientation with 150 bp insert and 50 bp standard deviation. For Illumina sequencing, forward and reverse reads from demultiplexed runs were 3'-end trimmed for nucleotides of quality <20 using the sickle algorithm (https://github.com/najoshi/sickle) and subjected to additional quality control using the Fast QC program (http://www.bioinformatics.babraham.ac.uk/projects/fastqc). Reads were aligned to the human genome (GATK repository, build 37) using BWA [39]. BAM files corresponding to single sequencing runs were subjected to PCR duplicate removal. Realignment around insertions/deletions and base recalibration was subsequently performed using GATK tools [40] release 2.2. BAM files for tumour and normal samples at this stage were used for obtaining alignment statistics and computing depth of coverage on-target (GATK tools). On-target BAM files were obtained by intersecting the base-recalibrated BAMs with the Illumina Truseq BED file.

Somatic variant calling was performed using MuTect2 [41] release 3.5 on tumour and normal on-target BAM files obtained for each patient. Unique variants with filter flag ‘PASS’ were collected from each sample and evaluated again using GATK UnifiedGenotyper. Among those, we selected variants that were not detected in all normal samples with more than two reads and were supported by at least either four or 10 reads in any of the tumours, for samples sequenced using SOLID or Illumina, respectively. Variants’ deleteriousness was evaluated using the CADD on-line service [42] and, to identify the most interesting variants, only mutations with a CADD score ≥4 were considered. Variant annotation was performed using the Variant Effect Predictor online tool (<http://www.ensembl.org/Tools/VEP>) [43] and the web-based platform Galaxy for data intensive biomedical research (https://usegalaxy.org). Finally, variants were further selected by visual inspection in the Integrative Genomics Viewer (IGV) [44].

**Targeted sequencing and copy number analysis**

Data analysis, including alignment to the GRCh37/hg19 human reference genome and variant calling, was carried out using the Torrent Suite Software v4.6 (Thermo Fisher Scientific, Milan, Italy). Filtered variants were annotated using a custom pipeline based on vcflib (https://github.com/ekg/vcflib), SnpSift [45], the Variant Effect Predictor (VEP) software [43] and NCBI RefSeq database. Alignments were visually verified with the IGV [44]. Targeted sequencing data were also used to estimate CNVs using the AmpliSeq Comprehensive Cancer Panel tumour–normal pair pipeline of the Ion Reporter Software, version 5.0 (Thermo Fisher Scientific). CNVs were detected by comparing matched tumour/normal BAM files; reported CNVs were filtered based on scores assigned by the software and visually verified with the IGV [44]. Pathway enrichment annotation for altered genes was performed using the gProfiler software [46] and the KEGG database (http://www.genome.jp/kegg/).

**Sanger sequencing**

A subset of somatic mutations identified through WES and targeted sequencing was validated using an orthogonal approach. Specific primers were designed (http://primer3.ut.ee) to amplify exon 3 of *CTNNB1*, exon 20 of *KDM6A*, exon 4 of *BAP1*, exon 4 of *TET1* and exon 9 of *SMAD4*. Primer sequences are available upon request. PCR products were sequenced using the 3130XL Genetic Analyzer (Thermo Fisher Scientific) and visualised using Mutation surveyor software (SoftGenetics, State College, PA, USA).
