# Supplementary material for: Molecular alterations associated with metastases of solid pseudopapillary neoplasms of the pancreas
Source: J Pathol. 2018 Nov 27;247(1):123–34. doi: 10.1002/path.5180 (PMC6588017; doi:10.1002/path.5180)
Supplement: Supplementary file 2 — Supplementary figure legends [file PATH-247-123-s002.docx]

**Molecular alterations associated with metastases of solid pseudopapillary neoplasms of the pancreas**

Amato E *et al*. *J Pathol* 2018 (DOI: 10.1002/path.5180)

**Supplementary figure legends**

**Figure S1. Flow charts of the sequencing analysis conducted on 15~~4~~ SPN cases.** The charts show the workflow of experiments conducted on 10 primary SPNs (A) and 5~~4~~ metastatic SPNs (B).

**Figure S2. Histological appearance of SPNs.** Haematoxylin and eosin staining of three representative tumours (scale bar = 100 µm; original magnification ×20). The microscopic features are heterogeneous within the same tumour showing a combination of solid and cystic components. The solid components consist of (A) pseudopapillae with vascular stalks and (B) hyalinised stroma within tumour areas. (C) A metastatic primary tumour showing a diffuse solid growth pattern with minimal supporting fibrovascular stroma.

**Figure S3. Histological characteristics of metastatic SPN**. Haematoxylin and eosin staining of the primary tumour (SPN13) and three metastatic lesions in the liver (SPN13_La; SPN13_Lb; SPN13_Lc; scale bar = 100 µm). Primary tumour shows morphological features suggestive of an aggressive behaviour, including mitosis (black arrowhead) and mild cellular atypia. For SPN13_La and SPN13-Lc, a dashed line separates the metastatic component (left) from the hepatic parenchyma (right). The hepatic lesion identified as SPN13-Lb presented with a distinct morphological component having sarcomatoid aspects (at the left of the dashed line).

**Figure S4. IHC staining for BTD, TP53, KDM6A and BAP1.** (A) No immunoreactivity was observed for BTD in normal pancreas, primary SPN and liver metastases. Positive staining for BTD was observed in endothelial cells and normal liver that were used as internal positive controls. (B) Immunostaining of p53 in TP53 wild type SPN and a metastatic SPN (SPN11) bearing a low allele frequency mutation of the *TP53* gene. (C) Representative IHC staining of normal pancreatic tissues for KDM6A (left) and BAP1 (right). Scale bars = 100μm; inset magnification 600×.

**Figure S5. Detail of gene-level somatic copy-number changes in five metastatic SPNs of the pancreas.** The matrix shows the chromosomal location and copy number status of genes as detected by high coverage targeted sequencing of 409 genes in five cases via matched analysis of normal, primary tumour and metastases. In one specimen (SPN11), LOH at chromosome 22 was detected through analysis of the allelic frequencies of SNPs in the affected genes. Alterations are annotated according to the following colour codes: light blue = LOH; light red = 3–5 copy gain; dark red >6 copy gain. See supplementary material, Table S5 for details.

**Figure S6. SLC2A1 expression is upregulated in PDAC bearing alterations of KDM6A.**

(A) Box plot showing the hypoxia signature score stratified by *KDM6A* status in the ICGC–PDAC cohort. ***p* < 0.01 as determined by Wilcoxon rank-sum test. Inactivation comprises mutation and structural variations. (B) Box plot showing the expression of *SCL2A1* in the TCGA–PDAC cohort in samples stratified based on *KDM6A* CNV. **p* < 0.05; ****p* < 0.001 by Wilcoxon rank-sum test. (C) Box plot showing the expression of *SCL2A1* in the ICGC–PDAC cohort in samples stratified based on *KDM6A* inactivation. **p* < 0.05 by Wilcoxon rank-sum test.
