# Supplementary figures and images for: Molecular alterations associated with metastases of solid pseudopapillary neoplasms of the pancreas
Source: J Pathol. 2018 Nov 27;247(1):123–34. doi: 10.1002/path.5180 (PMC6588017; doi:10.1002/path.5180)

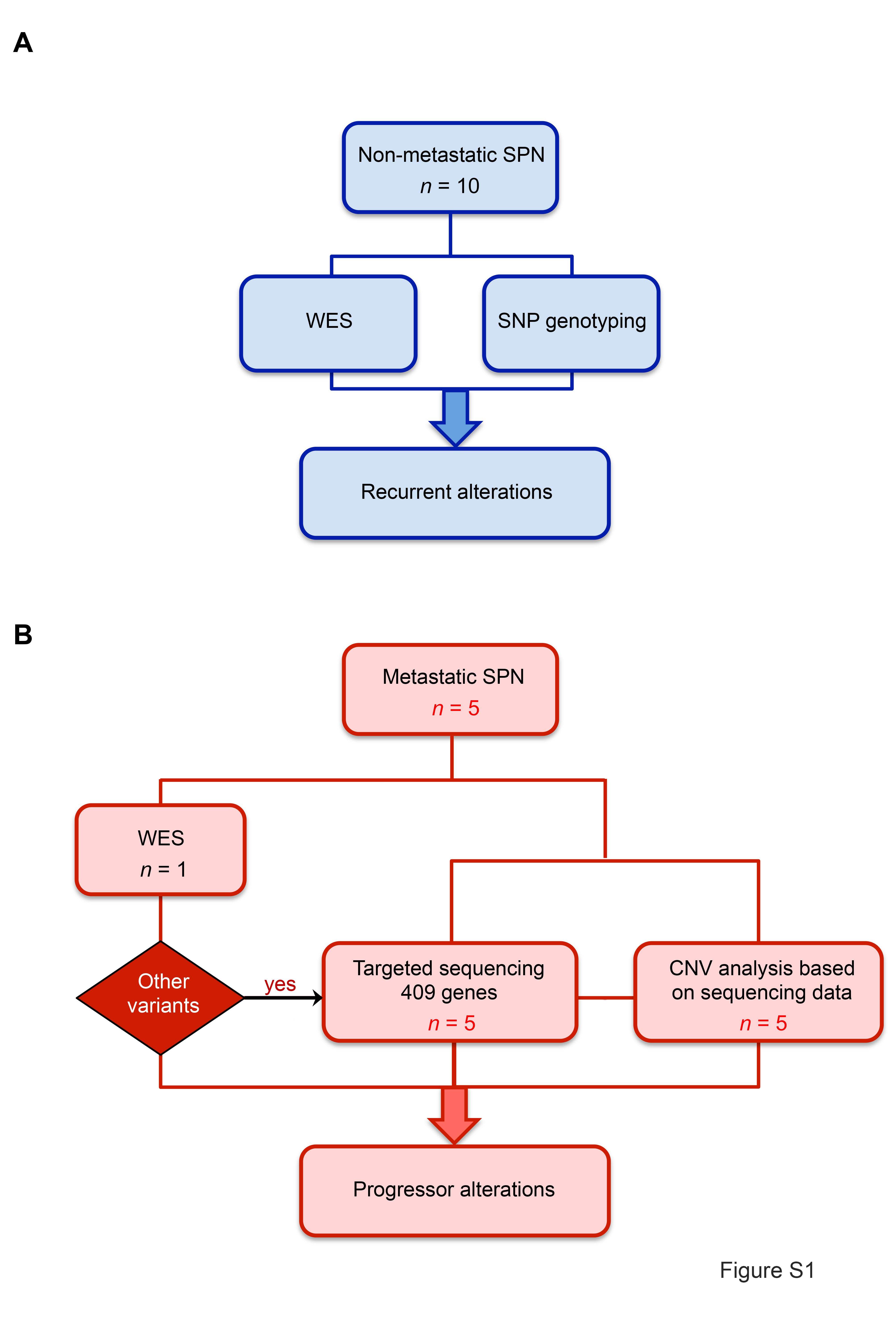

Supplement: Supplementary file 3 — Figure S1. Flow charts of the sequencing analysis conducted on 154 SPN cases [file PATH-247-123-s003.tif]

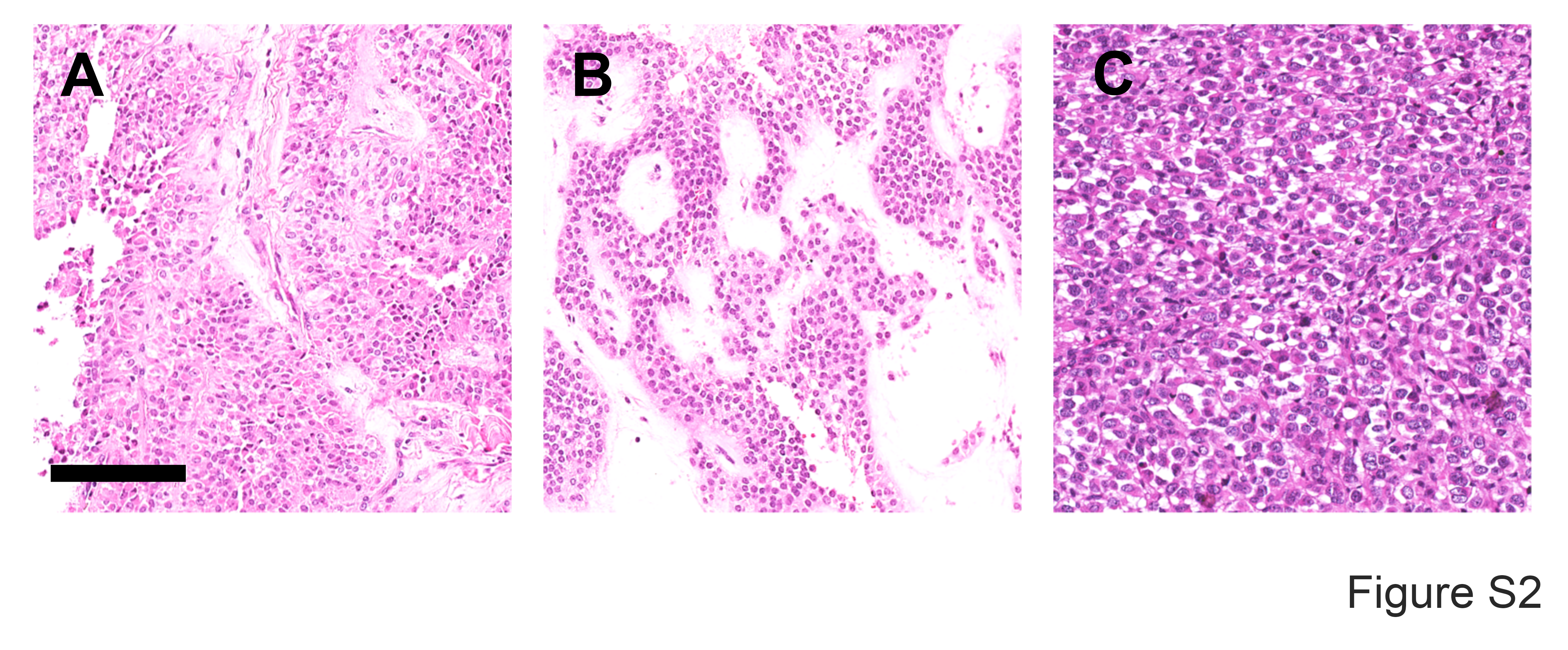

Supplement: Supplementary file 4 — Figure S2. Histological appearance of SPNs [file PATH-247-123-s004.tif]

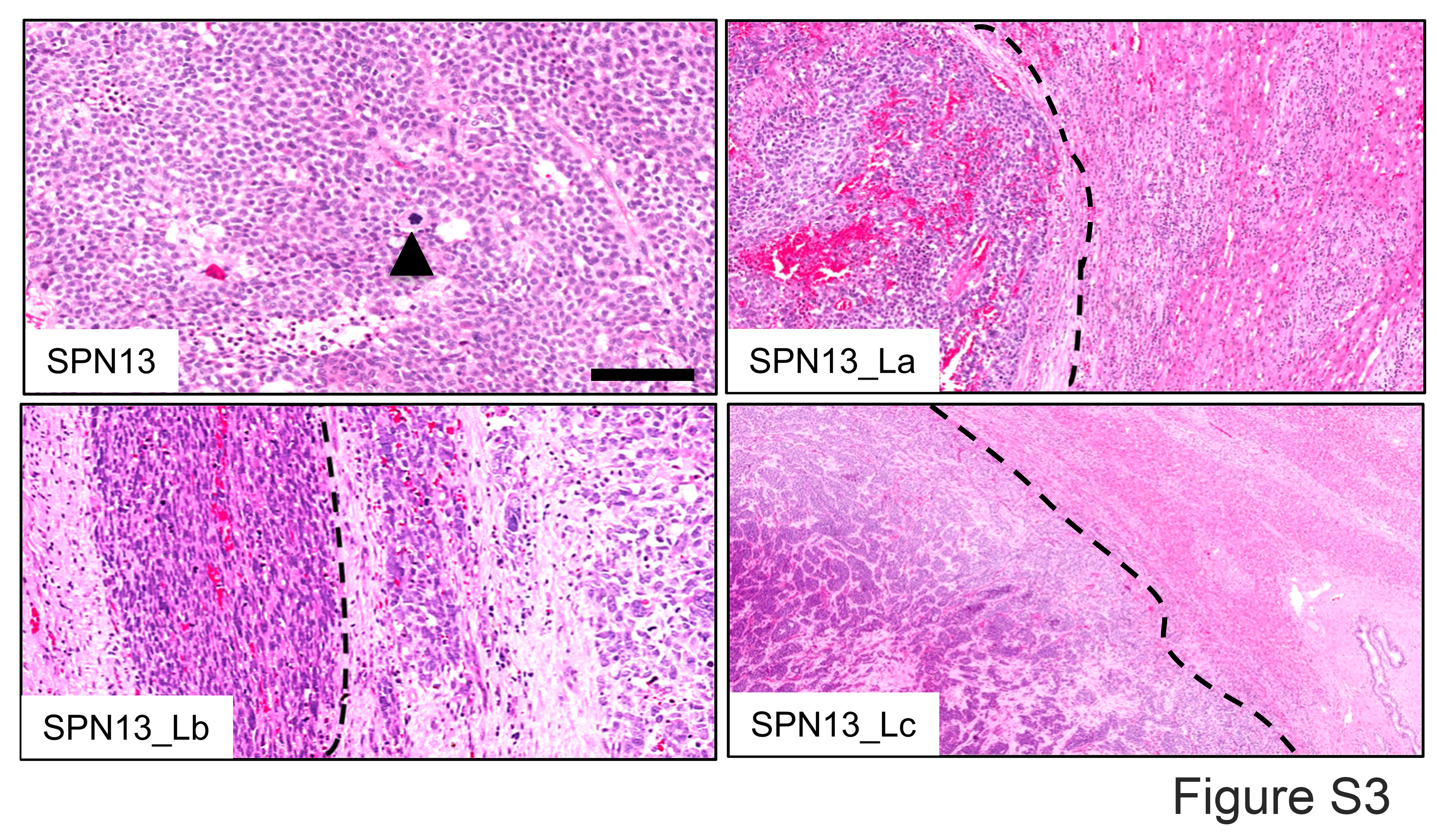

Supplement: Supplementary file 5 — Figure S3. Histological characteristics of metastatic SPN [file PATH-247-123-s005.tif]

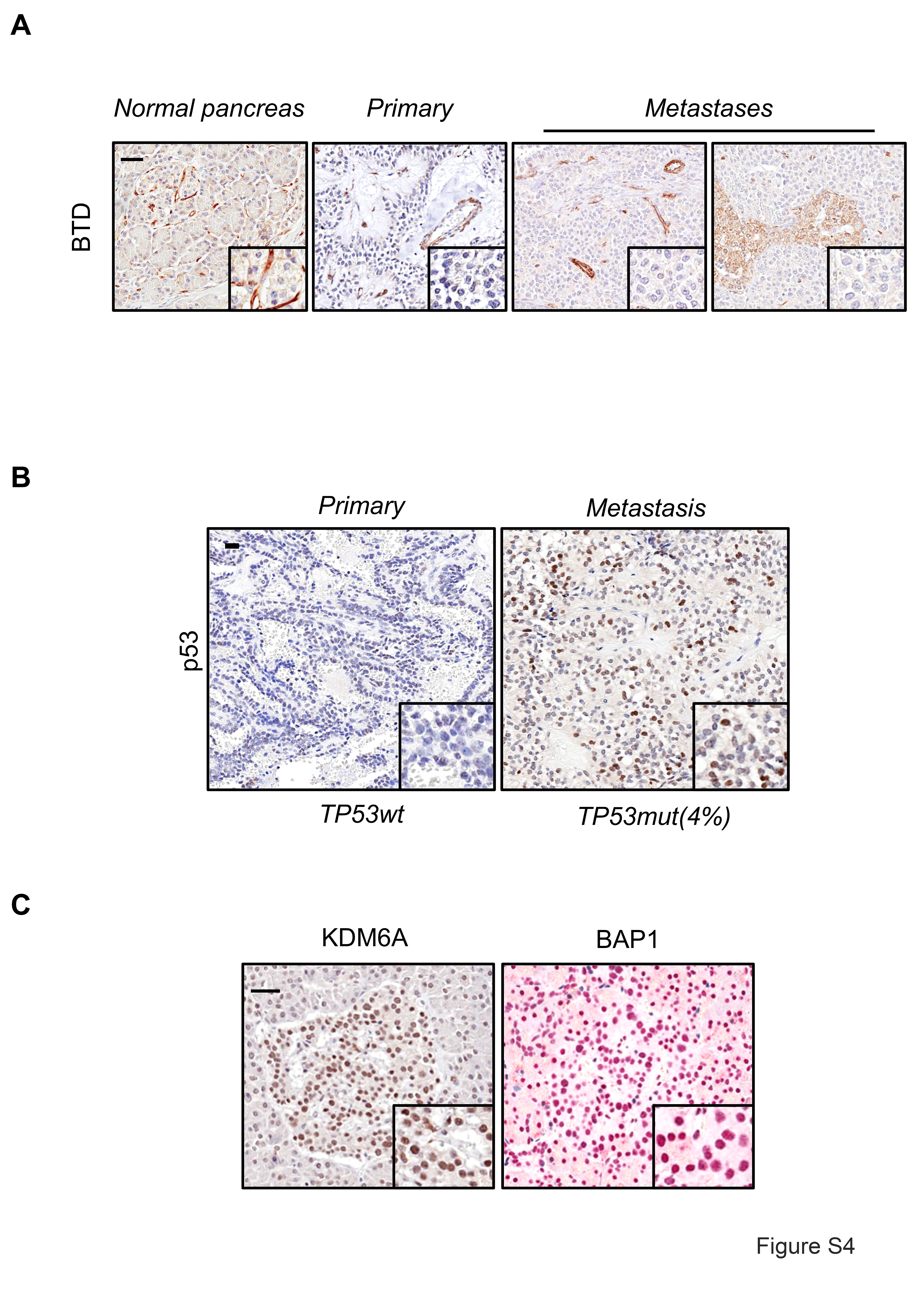

Supplement: Supplementary file 6 — Figure S4. IHC staining for BTD, TP53, KDM6A and BAP1 [file PATH-247-123-s006.tif]

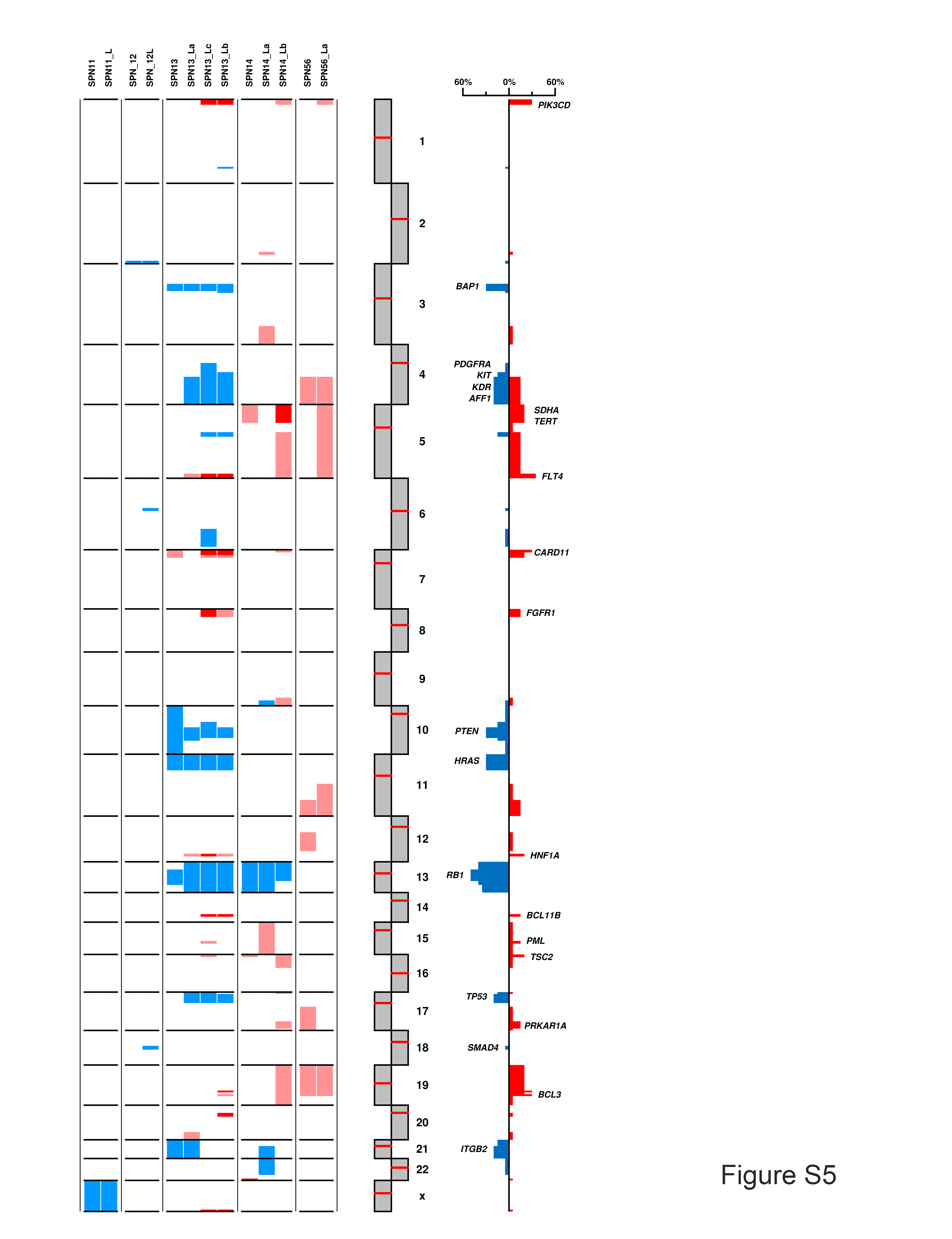

Supplement: Supplementary file 7 — Figure S5. Detail of gene‐level somatic copy‐number changes in five metastatic SPNs of the pancreas [file PATH-247-123-s007.tif]

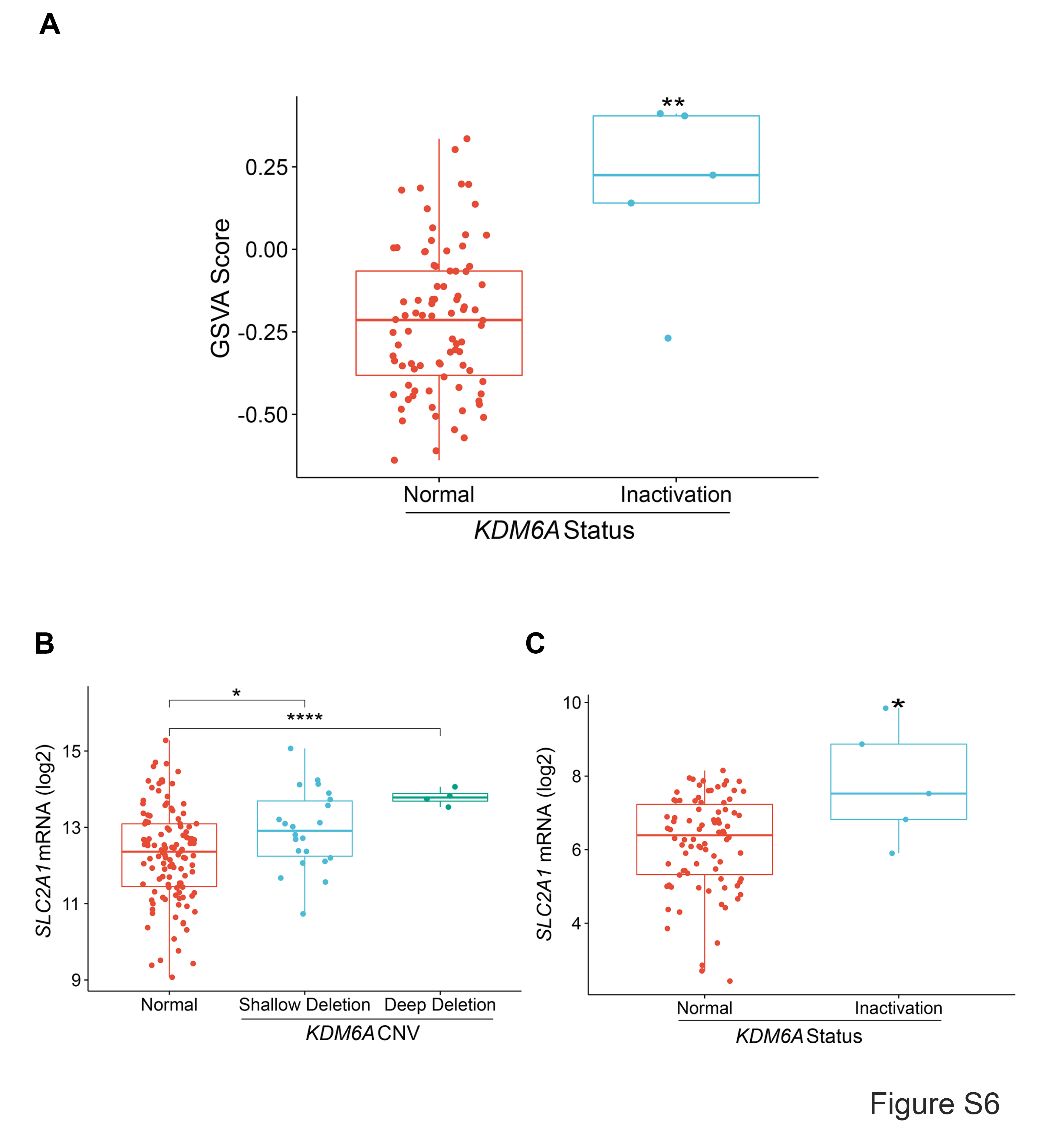

Supplement: Supplementary file 8 — Figure S6. SLC2A1 expression is upregulated in PDAC bearing alterations of KDM6A [file PATH-247-123-s008.tif]
